# Downregulation of IRS-1 in adipose tissue of offspring of obese mice is programmed cell-autonomously through post-transcriptional mechanisms^☆^

**DOI:** 10.1016/j.molmet.2014.01.007

**Published:** 2014-01-20

**Authors:** Denise S. Fernandez-Twinn, Maria Z. Alfaradhi, Malgorzata S. Martin-Gronert, Daniella E. Duque-Guimaraes, Ana Piekarz, David Ferland-McCollough, Martin Bushell, Susan E. Ozanne

**Affiliations:** 1 University of Cambridge Metabolic Research Laboratories and MRC Metabolic Diseases Unit, Institute of Metabolic Science, Level 4, Box 289, Addenbrooke's Hospital, Cambridge CB2 0QQ, UK; 2 MRC Toxicology Unit, Hodgkin Building, PO Box 138, University of Leicester, Lancaster Road, Leicester LE1 9HN, UK

**Keywords:** DEXA, dual energy X-ray absorptiometry, IRβ, insulin receptor-beta, miRNA, microRNA, PI3K, phosphotidylinositol 3-kinase, UTR, untranslated region, Maternal obesity, Adipose tissue, Hyperinsulinemia, IRS1, microRNAs

## Abstract

We determined the effects of maternal diet-induced obesity on offspring adipose tissue insulin signalling and miRNA expression in the aetiology of insulin resistance in later life. Although body composition and glucose tolerance of 8-week-old male offspring of obese dams were not dysregulated, serum insulin was significantly (*p*<0.05) elevated. Key insulin signalling proteins in adipose tissue were down-regulated, including the insulin receptor, catalytic (p110β) and regulatory (p85α) subunits of PI3K as well as AKT1 and 2 (all *p*<0.05). The largest reduction observed was in IRS-1 protein (*p*<0.001), which was regulated post-transcriptionally. Concurrently, miR-126, which targets IRS-1, was up-regulated (*p*<0.05). These two features were maintained in isolated primary pre-adipocytes and differentiated adipocytes *in-vitro*. We have therefore established that maternal diet-induced obesity programs adipose tissue insulin resistance. We hypothesise that maintenance of the phenotype *in-vitro* strongly suggests that this mechanism is cell autonomous and may drive insulin resistance in later life.

## Graphical abstract

**Figure:**
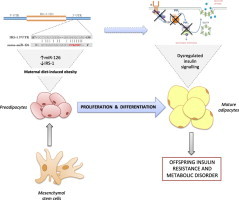

## 1 Introduction

The prevalence of obesity has been increasing at alarming rates in both the developed and the developing world [1]. Of particular concern are the increasing rates of obesity in women of childbearing age [2]. In the USA the prevalence of obese women aged 20–39 years old tripled between 1960 and 2000 [3] and in the UK, between 1990 and 2004, the BMI of pregnant women at their first prenatal booking increased by an average of 1.37 kg/m^2^ [4]. These statistics have raised concern as epidemiological studies have revealed a relationship between maternal obesity and risk of obesity and other features of the metabolic syndrome in her offspring (reviewed in Alfaradhi and Ozanne [5]). Although the inheritance of susceptibility genes may contribute to this association, growing evidence from studies in both humans and animal models suggests that exposure to an obesogenic environment *in utero* plays an important role in mediating this relationship. In humans some of the best evidence in support of such programmed effects has come from the study of siblings born to the same mother before and after she had bariatric surgery to reduce her weight. These demonstrated a reduced risk of obesity and other adverse effects in the offspring born after the surgery compared to those born before surgery [6,7]. These findings support the notion that exposure to an obesogenic environment *in utero* is able to developmentally programme the offspring physiology. It is therefore likely that increased maternal obesity during pregnancy is contributing to the alarming rise in the prevalence of childhood obesity and type-2 diabetes in the next and subsequent generations.

Animal models strongly support the concept that maternal obesity during pregnancy can transmit to the offspring, through non-genetic mechanisms, an increased risk of obesity, insulin resistance and glucose intolerance (reviewed in [5]). These include models in non-human primates [8,9], sheep [10], maternal high fat fed rodents [11–13], and rodent models employing a cafeteria diet [14,15]. Our recent studies have focused on a mouse model of maternal diet-induced obesity using a diet rich in fat and simple sugars that is representative of a western human diet. Offspring of dams fed this diet throughout pregnancy and lactation gain excess weight, become obese, insulin resistant and develop impaired glucose tolerance in adulthood [16]. In addition they develop non-alcoholic fatty liver disease and demonstrate muscle mitochondrial dysfunction [17]. These differences are associated with reduced expression of the insulin receptor (IR), insulin receptor substrate-1 (IRS-1) and the p85α regulatory subunit of phosphatidylinositol 3-kinase (PI3K) regulatory subunit in the liver [17,18] and reduced expression of the p110β catalytic subunit of PI3K in skeletal muscle [19]. However, as these parameters have been measured at a time point when the animals are obese, it is not clear which of these are consequences of offspring obesity and which arise as a direct effect of maternal obesity independently of offspring obesity.

Studies in low birth weight humans and animal models of under-nutrition have demonstrated that insulin resistance can be a primary consequence of a suboptimal early environment and can arise independently of changes in offspring obesity [20–24]. The insulin resistance is associated with changes in expression of key insulin signalling proteins [22,23]. In humans and rodents some of the largest effects on insulin signalling protein expression are observed in adipose tissue, an organ whose importance in regulation of whole body glucose homoeostasis has become increasingly apparent in recent years [25–27]. IRS-1 and PI3K proteins appear to be some of the most vulnerable to the effects of early under-nutrition [22].

The aim of the current study was to determine whether changes in insulin sensitivity resulting from maternal diet-induced obesity precede development of obesity in the offspring and if this was associated with changes in adipose tissue insulin signalling protein expression. We further aimed to investigate the potential mechanisms underlying the programming of insulin signalling protein expression with a focus on post-transcriptional gene regulation.

## 2 Materials and methods

### 2.1 Animal model

All studies were approved by the Local Ethics Committee and were conducted according to the Home Office Animals (Scientific Procedures, UK) Act 1986. The model has been described in detail previously [18]. Briefly, female C57BL/6J mice, approximately 4 weeks of age were fed ad libitum either a standard control chow (7% simple sugars, 3% fat [w/w]) RM1 diet or a highly palatable energy-rich obesogenic diet (10% simple sugars, 20% animal fat [w/w]) and sweetened condensed milk (55% simple sugar, 8% fat, 8% protein [w/w], Nestle (Nestle, UK) fortified with mineral and vitamin mix AIN93G), for 6 weeks before mating for first pregnancy. Both diets were purchased from Special Dietary Services, Witham UK, compositions of which have been previously described [28]. The dams were allowed to litter and the first litter culled after weaning. This first pregnancy ensured the mice were proven breeders. After a week, mice were re-mated for a second pregnancy and day 1 of pregnancy was defined by the appearance of a plug. Dams were maintained on their respective experimental diets throughout both pregnancies and lactation periods and were weighed at the beginning and end of pregnancy. Litter sizes were standardized to 3 males and 3 females on postnatal day 3. Male offspring only were used in this study. The experimental offspring groups [Control and Maternal obesity (Mat-Ob); *n*=8 liters per group] were weaned onto standard chow (RM1) at 21 days of age and remained on this diet until the end of the study. Body weights and food intake was recorded weekly. At 8 weeks of age, following an overnight fast offspring were killed by raising CO_2_ concentration. Blood was taken for serum biochemical analyses. Adipose tissue, liver, vastus lateralis muscle and heart tissue were dissected, weighed, snap frozen and stored at −80 °C until use.

### 2.2 Dual-energy X-ray absorptiometry measurement of body composition

Body composition analysis was performed by dual energy X-ray absorptiometry (DEXA, Lunar PIXImus densitometer; GE Lunar Corp., Madison, WI) on dams at weaning and on offspring at 8 weeks of age immediately after killing by CO_2_ asphyxiation.

### 2.3 Indirect calorimetry

Oxygen consumption (VO_2_), activity (horizontal, vertical, and ambulating) and food intake were measured using an Oxymax open-circuit indirect calorimetry system (Columbus Instruments International, Columbus, OH). Following a training period the week before, male mice were placed in calorimeter chambers for 72 h in a light- (12-h light, 12-h dark cycle) and temperature-controlled environment with free access to standard chow and water throughout the duration of the 72-h measurement period. Energy expenditure was recorded as J/min in 2 hourly intervals and activity data is reported as mean counts of horizontal activity (X-tot), ambulation (X-amb) and vertical activity (Z-tot). Food intake data is reported as the average daily intake during calorimetry.

### 2.4 Plasma analysis

Fasted glucose concentrations were determined in tail blood using a blood glucose analyser (AlphaTRAK glucose meter, Abbot Logistics B.V., Netherlands). Insulin was measured using a Meso Scale Discovery kit (Gaithersburg, MD, USA). Triglycerides, free fatty acids and cholesterol were measured using an enzymatic assay (Dade Behring, Siemens Healthcare). All analyses were carried out on serum collected after an overnight fast (Core Biochemical Assay Laboratory, Cambridge University Hospitals NHS Foundation Cambridge, UK).

### 2.5 Glucose tolerance test (GTT)

Mice were fasted overnight and blood was drawn from the tail for basal glucose measurements (AlphaTRAK, Abbot Logistics B.V., Netherlands). Mice were then injected intraperitoneally (i.p.) with 1 g/kg glucose. Further blood glucose measurements were made at timed intervals. AUC was calculated by summation of trapezoids (Prism, GraphPad, La Jolla, USA).

### 2.6 Western blotting

Adipose tissue samples were homogenised in lysis buffer [50 mmol/l HEPES (pH 8), 150 mmol/l sodium chloride, 1% Triton X100, 1 mmol/l sodium orthovanadate, 30 mmol/l sodium fluoride, 10 mmol/l sodium pyrophosphate, 10 mmol/l EDTA and a protease inhibitor cocktail (set III, Calbiochem Novabiochem Biosciences, Nottingham, UK)] and total protein concentration was measured using a copper/bicinchoninic assay (Sigma-Aldrich, UK). Samples (20 µg protein) were subjected to SDS-PAGE and transferred onto a PVDF membrane (Immobilon-P, Millipore, Billerica, MA, USA). Following blocking (in 5% non-fat dehydrated milk, 1× TBS, 0.1% Tween 20), the membrane was incubated overnight with the primary antibody [IRS-1 and PI3K p85α subunit (Upstate Biotechnology, Millipore, Lake Placid, USA), PI3K p110β subunit, AKT1, AKT2 and, (Cell Signalling, New England Biolabs, UK); IRβ and protein kinase C-ζ (PKCζ) (Santa Cruz Biotechnology, Heidelberg, Germany)]. Following washing with TBS/T (1× TBS, 0.1% Tween 20) membranes were incubated with horseradish peroxidase-conjugated anti-rabbit or anti-mouse antibody (Jackson ImmunoResearch, Stratech, UK). Antibody binding was detected using Super Signal West Pico Chemiluminescent substrate (Thermo Scientific, UK) and audioradiographed images were quantified using AlphaEase software (AlphaInnotech, San Leandro, USA). Twenty microgram and 10 µg of a pooled sample was loaded in each gel and a correct ratio confirmed linearity of signal for each antibody.

### 2.7 Gene expression

Total RNA was extracted from adipose tissue samples using the *mir*VANA miRNA Isolation Kit (Ambion Inc., UK). cDNA was generated using a High-Capacity cDNA Reverse Transcription Kit (Applied Biosystems, UK) and quantitative real time PCR (qPCR) performed on a StepOnePlus™ Real-Time PCR System (Applied Biosystems). For SYBR green assays the primer mix per well consisted of 400 nM primer (Sigma-Aldrich, UK). For primer sequences see (Supplementary Table 1), 1× SYBR Green master mix (Applied Biosystems, UK) and a 1:10 dilution of sample cDNA. A standard curve was generated using serial dilution of the pooled cDNA samples. mRNA expression was normalised to the geomean of selected housekeeping genes from beta-actin, cyclophilin, PABP1 and HPRT (as stated in figures). For Taqman assays (Applied Biosystems, UK) the primer mix per well consisted of 1× Taqman primer (Egfl7 assay ID: Mm00618004_m1), 1× Taqman PCR master mix and 100 ng template cDNA. A standard curve was generated as above and expression was normalised to beta-actin (Assay ID: Mm00607939_s1).

### 2.8 microRNA expression

Total RNA was reversed transcribed using miR-specific primers (TaqMan MicroRNA Assays) and Taqman MicroRNA Reverse Transcriptase Kit (Applied Biosystems, UK) followed by qPCR quantification with Taqman 2x Universal PCR Master Mix No AmpErase UNG (Applied Biosystems, UK). For miR-126, this was hsa-*miR-126* Assay ID:002228 (Applied Biosystems, UK). Standard curves were generated as above and microRNA expression was normalised to the geomean of housekeepers including miR-185 (Assay ID: 002271, Applied Biosystems, UK) and U6 snRNA (Assay ID: 001973, Applied Biosystems, UK) as well as sno25, U5A and sca17 (miScript primer assays, Qiagen, Manchester, UK), none of which differed between groups.

### 2.9 Luciferase reporter constructs and assay

Luciferase reporter constructs were generated by cloning the 3'-UTR portion of rat IRS-1 mRNA containing the seed target sequence of miR-126. PCR on rat white adipose tissue cDNA yielded 1068 of 1086 bp of mouse 3'-UTR. The PCR product was then sub-cloned downstream of the luciferase gene contained in the pGL3-Basic commercial vector (Promega, Southampton, UK).

HeLa cells were used for miR-126 over expression studies, and HEK293 cells for miR-126 antagonism studies. Cells were transfected with 100 ng of Firefly-luciferase reporter construct with 10 ng of Renilla-luciferase pRL-SV40 (Clontech, Basingstoke, UK) as a transfection control and increasing concentrations of hsa-miR-126 mimic (Life Technologies, CA, USA) (0, 10, 50 and 100 nM in HeLa) or miR-126 2'-O-Methyl antagonist (Sigma-Aldrich, Dorset, England) (0, 10, 50 and 100 nM in HEK293) using RNAi Max transfection agent (Invitrogen, Paisley, UK). Cells were harvested and the luciferase assay performed using LAR-II substrate (Promega). Luminescence was detected using the GloMax system (Promega) and data is presented as a F-Luc signal over R-Luc signal. The primer sequences for 3'UTR cloning of rat IRS-1 were: Forward primer-CTTAACTGGACGTCACAGGCAGAAT and Reverse primer-CCGGGGGAAAGGCTTATAGAAGTGG. The 2'-O-Methyl rno-miR-126 antagonist sequence used was CGCAUUAUUACUCACGGUACGA, where every base contained a 2'-O-Methyl modification.

### 2.10 Isolation of primary preadipocytes and differentiation in-vitro

Following killing by carbon dioxide asphyxiation, epididymal fat was dissected from 8-week-old male mice and collected into fresh Hanks Basic Salt Solution (HBSS). The tissue was then minced with scissors and subjected to controlled collagenase digestion in 10 ml of Digestion solution (15 mg Collagenase Type II, 225 mg bovine serum albumin in HBSS) (all chemicals from Sigma Aldrich, Poole, UK) with shaking for 30–40 min at 37 °C and 180 rpm till a homogenous digest was obtained. The solution was then strained through a 100 μm mesh (BD Falcon, Corning, Tewksbury, USA) and moved to ice for 20 min. The upper white layer (mature adipocytes) were removed and the next two-thirds of the digest was taken and mixed with growth medium (High glucose Dulbecco's modified Eagle Medium supplemented with 10% newborn calf serum, 1% Penicillin-Streptomycin, 200 μM l-glutamine). This stromal-vascular fraction was then centrifuged twice, resuspending in growth medium each time. After the final spin, cells were counted and plated at a density of 2×10^5^ per well onto 6 well plates. Cells that were to be differentiated were supplemented with an adipogenic cocktail of 10 μM insulin (Actrapid) and 150 μM sodium ascorbate, daily for 11 days (modified from Shimizu et al. [29]) while undifferentiated cells were maintained for 11 days in growth medium without adipogenic cocktail. Cells were harvested on day 11.

### 2.11 IRS-1 decay following translational block

Primary preadipocytes were cultured for 10 days before treating with cycloheximide (100 μmol) for 24 h. Cells were then lysed immediately in lysis buffer (see Section 2.6). Protein concentrations in lysates were quantified (BCA kit, Sigma Aldrich, Poole UK), and then standardised to a common concentration before equal amounts of protein were loaded and analysed by Western blotting for IRS-1.

### 2.12 Statistical analysis

Data were analysed from one male per litter hence ‘*n*’ refers to the number of litters per group. Data were analysed by ANOVA using Prism 6 (Graphpad, La Jolla, USA). Protein expression data are presented as percentage mean expression of the control group±SEM. All other data is shown as mean±SEM. For all data, *p*<0.05 was considered statistically significant.

## 3 Results

### 3.1 Maternal phenotype

#### 3.1.1 Body composition

Feeding mice an obesogenic diet resulted in significantly increased body weight at the start of pregnancy compared to mice fed a control diet (32.7±0.8 g vs. 28.5±0.4 g; *p*<0.001, *n*=6). Dams fed the obesogenic diet were also significantly heavier than control mothers at the end of lactation (38.0±1.1 g vs. 31.1±0.6 g; *p*<0.05, *n*=8). This was accompanied by increased calorie intake in the obese mothers during pregnancy (110.8±14.0 vs. 65.3±2.2 kJ per day; *p*<0.05, *n*=8) and lactation (217.6±6.6 vs. 140.9±8.0 kJ per day; *p*<0.0001, *n*=8). Body composition of the mothers, assessed by DEXA at the end of lactation, showed that obese mothers had twice as much total body fat mass as control dams both in absolute terms and as a percentage of body weight, while absolute lean mass remained constant (Table 1).

#### 3.1.2 Plasma analysis

Circulating glucose levels in dams at weaning were elevated in the fed state in obese mothers compared to controls *p*<0.05 (Table 2). Levels of free fatty acids (*p*<0.05), total cholesterol (*p*<0.001) and HDL cholesterol (*p*<0.001) were all significantly raised in obese mothers compared to controls. Triglyceride levels were unchanged.

### 3.2 Offspring phenotype

#### 3.2.1 Body composition

At 8 weeks of age, offspring of obese dams were a similar weight to control offspring. Lean mass and fat mass, as assessed by DEXA, were also similar between the two experimental groups. Bone density was also comparable at this age (Supplementary Table 2).

#### 3.2.2 Metabolic phenotyping

**Figure 1:**
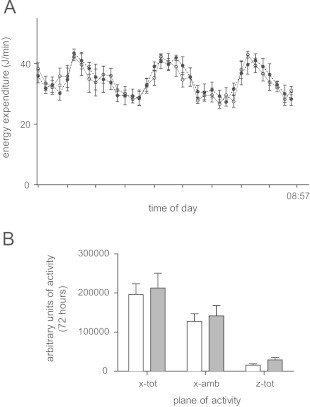
Energy expenditure and activity at 8 weeks of age. (A) Energy expenditure of Control (white circle, solid line) and Mat-Ob (black circle, dashed line) offspring. Mice previously trained 1 week prior to the procedure, were singly housed for a 72 h period in a CLAMS chamber with free access to both food and water. Parameters of oxygen and carbon dioxide exchange coupled to food intake enabled quantification of both RER and energy expenditure. No significant differences determined by repeated measures ANOVA. (B) Physical activity as mean counts of horizontal activity (x-tot), ambulation (x-amb) and vertical activity (z-tot) in the Control (white bars, *n*=6) and Mat-Ob (grey bars, *n*=5) groups. No significant differences determined by a 2-way ANOVA.

**Figure 2:**
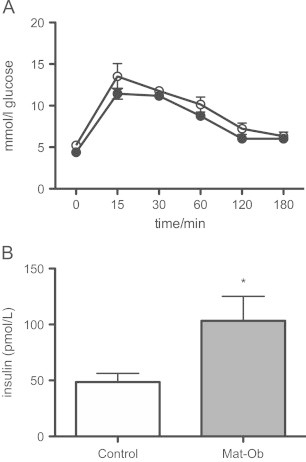
(A) Glucose tolerance following 1 g/kg body weight bolus i.p. glucose injection in Control (open circles) vs. Mat-Ob (closed circles) offspring; and (B) fasting serum insulin concentrations in Control (white bars) and Mat-Ob (grey bars) offspring. *n*=6 per group. ^⁎^=*p*<0.05 determined by student's *t*-test.

Energy expenditure measured over 72 h was not significantly different between the two experimental groups (Figure 1A). Activity levels were also not significantly different between the two groups (Figure 1B). At 8 weeks of age, glucose tolerance was similar between the two groups (Figure 2A). However, fasting insulin levels were significantly higher in the offspring of obese mothers compared to controls (Figure 2B).

#### 3.2.3 Insulin signalling protein expression in epididymal fat

**Figure 3:**
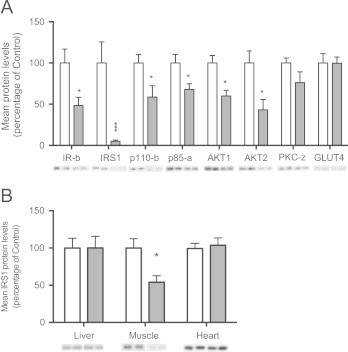
(A) Protein levels of insulin signalling molecules in adipose tissue at 8 weeks of age. (B) Protein levels of IRS-1 in liver, muscle and heart tissue of 8-week-old males. Signal intensities were quantified from *n*=6 per group, Control (white bars) and Mat-Ob (grey bars) and data presented as percentage mean expression of the control group±SEM. ^⁎^=*p*<0.05, ^⁎⁎⁎^=*p*<0.001 determined by student's *t*-test.

Protein expression of IRβ was down regulated in epididymal fat of the Mat-Ob group compared to controls (Figure 3A). Protein levels of downstream signalling molecules, including the p110β and p85α subunits of PI3K as well as AKT1 and AKT2 (all *p*<0.05), were also significantly reduced in the Mat-Ob group, whereas levels of PKCζ and GLUT4 were similar between the two groups (Figure 3A). However, the biggest difference observed was in IRS-1 that was reduced in the offspring of the obese dams (*p*<0.001) to less than 10% of control levels. We also observed a similar reduction in IRS-1 protein levels in the retroperitoneal fat depot (33±16 in the Mat-Ob group vs. 100±28 in the Control group; *p*<0.05, *n*=8). However, there were no differences in IRS-1 mRNA levels either in the epididymal fat depot (0.89±0.12 in the Mat-Ob group vs. 1.00±0.11 in the Control group) or the retroperitoneal fat depot (0.96±0.09 in the Mat-Ob group vs. 1.00±0.04 in the Control group). In order to test the tissue specific effects of the obesogenic maternal diet on IRS-1 protein levels, we also measured the protein expression of IRS-1 in liver, skeletal muscle and heart. Protein levels were similar between groups in the liver and heart. However, there was a significant reduction in IRS-1 protein expression in vastus lateralis in the Mat-Ob group compared to controls (Figure 3B).

#### 3.2.4 microRNA (miRNA) expression

**Figure 4:**
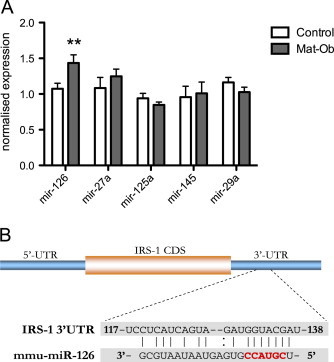
miRNA expression in adipose tissue of 8-week-old male offspring. (A) Expression of 5 candidate miRNAs relative to miR-185 and snRNA U6 housekeepers. (B) IRS-1 protein level and parallel miR-126 expression in adipose tissue from 8-week old male offspring. (C) Seed sequence of *miR-126* (red) predicted to target the *IRS1* 3'-UTR. *n*=8 per group, Control (white bars) and Mat-Ob (grey bars). ^⁎^*p*<0.05 determined by Student's *t*-test.

As the most marked effect of maternal diet-induced obesity was on IRS-1 protein expression without changes at the mRNA level, we chose to examine mechanisms underlying this effect in further detail focusing on the potential role of miRNAs. A candidate approach was adopted to investigate differences in miRNAs with complementary binding seed sequences in the 3'UTR of the *IRS1* gene. We investigated a panel of five miRNAs, which were selected based on bioinfomatic assessment of their putative binding to the 3'UTR of *IRS1*. Of these five miRNAs, only *miR-126* was differentially expressed in adipose tissue of the Mat-Ob group (Figure 4A). Whilst IRS-1 levels in adipose tissue are dramatically reduced in the Mat-Ob group, *miR-126* expression is elevated in parallel, suggesting that *miR-126* can regulate IRS-1 protein expression in adipose tissue. The *miR-126* primary sequence is located within an intron of *Egfl7*. To determine if Eglf7 mRNA was elevated concomitantly with *miR-126*, we measured Egfl7 mRNA expression. However expression of *Egfl7* was comparable between the two experimental groups (1.04±0.07 in the control group vs. 0.94±0.15 in the Mat-Ob group), which suggests that miR-126 is differentially regulated from its host gene.

**Figure 5:**
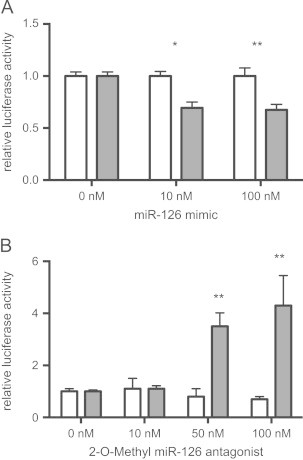
(A) Relative luciferase activity in control (white bars) constructs or constructs containing the 3' UTR of *IRS1* (grey bars) introduced into HeLa cells in the presence of miR-126 mimic. Firefly luciferase activity for each construct was normalised to the co-transfected Renilla luciferase construct and then normalised to the change in luciferase in the presence of miR-126 (normalised luciferase activity in the absence of miR-126 was set to 1). (B) Relative luciferase activity of the constructs used in (A) in HEK 293 cells upon inhibition of miR-126 with 2-O-Methyl miR-126 antagonist. Normalisation was performed as in (A). ^⁎⁎^*p*<0.01, ^⁎^*p*<0.05.

To confirm that *miR-126* mediates the repression of *IRS1* translation, the 3'-UTR of *IRS1* encompassing the *miR-126* target site (Figure 4B) was cloned in a luciferase-based reporter plasmid. The construct was co-transfected in HeLa cells with increasing concentration of *miR-126* mimic. The presence of exogenous *miR-126* resulted in a significant reduction in luciferase activity compared to the control (Figure 5A). Conversely, introduction of a *miR-126* antagonist in Hek293 cells resulted in increased luciferase output of a reporter construct under the control of the *IRS1* 3'-UTR (Figure 5B). This data therefore suggests a direct interaction between *miR-126* and its binding site in the 3'-UTR of *IRS1*.

#### 3.2.5 IRS-1 reduction and miR-126 overexpression is retained in isolated Mat-Ob primary preadipocytes differentiated in-vitro

**Figure 6:**
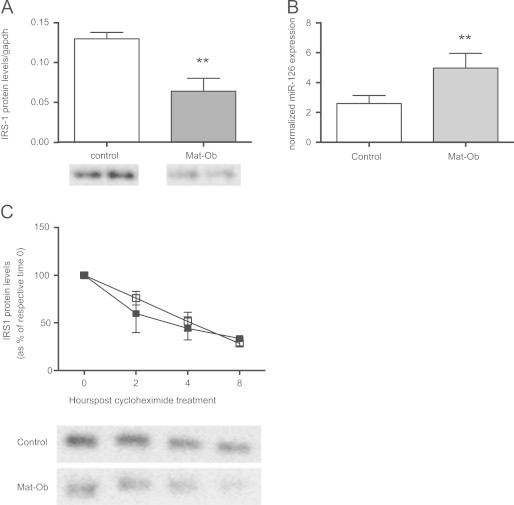
(A) IRS-1 protein levels and (B) miR-126 expression in adipocytes derived from primary cultures of preadipocytes and differentiated for 11 days *in-vitro* (from *n*=4 liters per group, Control (white bars) and Mat-Ob (grey bars)). ⁎⁎ *p*<0.01 determined by Student's *t*-test. (C) Adipocytes derived from primary cultures of preadipocytes [from *n*=4 liters per group, Control (open boxes) and Mat-Ob (closed boxes)] were cultured for 10 days before treatment with cycloheximide. IRS-1 protein was measured by Western blotting and expressed as a percentage of respective protein levels at time 0.

A reduction in IRS-1 protein levels was observed in Mat-Ob adipocytes derived from Mat-Ob preadipocytes that had been differentiated *in-vitro* compared to controls (Figure 6A). In parallel the increased expression of *miR-126* was also maintained in preadipocytes and differentiated adipocytes isolated from the Mat-Ob group compared to those isolated from control offspring (Figure 6B).

#### 3.2.6 Maternal obesity does not affect rate of IRS-1 degradation

To directly determine if maternal obesity had any effect on the rate of IRS-1 protein degradation *in-vitro*, primary preadipocytes were cultured for 10 days and then treated with cycloheximide to block translation. IRS-1 protein levels over time were detected by western blotting and the rate of protein degradation measured. No difference in the rate of IRS-1 decay was observed between the two groups (Figure 6C).

## 4 Discussion

In the current study we investigated animals in young adult life to dissect out the effects of maternal diet-induced obesity on offspring insulin resistance that were independent of the increased adiposity previously observed in older offspring [16]. We showed that at 8 weeks of age offspring of obese dams have similar body weights and body composition to the offspring of control-fed dams, as well as a similar glucose tolerance. However, the offspring of the obese dams were hyperinsulinaemic, suggesting that they required increased insulin concentrations to maintain normoglycaemia and were therefore insulin resistant. This suggests that, as has been shown in low birth weight humans and rodent models of under-nutrition, maternal over-nutrition can lead to insulin-resistance in the offspring that is not related to increased adiposity.

The three key tissues regulating glucose homoeostasis in response to insulin are the liver, skeletal muscle and the adipose tissue. Although skeletal muscle has been recognised for many years as the major site of glucose disposal postprandially, the importance of adipose tissue in maintaining euglycaemia has become increasingly apparent in recent years. For example, adipose tissue specific overexpression of GLUT4 was able to reverse insulin resistance and diabetes in mice selectively lacking muscle GLUT4 [30]. Furthermore, the fat-specific ablation of Rictor – a component of the mTOR complex mTORC2, which catalyses the phosphorylation of AKT – was found to impair whole body glucose and lipid metabolism [31]. The current study demonstrates that maternal diet-induced obesity leads to impaired adipose tissue insulin signalling in young mice of obese mothers through reduced IRβ, IRS-1 and the p110β catalytic and p85α regulatory subunits of PI3K. We postulate that this contributes to the observed peripheral insulin resistance. It is known that chronic hyperinsulinaemia exacerbates insulin resistance and contributes to β-cell failure and ultimately leads to type-2 diabetes [25]. Insulin resistance in the adipose tissue, leading to increased demands on pancreatic β-cells to produce insulin could therefore contribute to the eventual hypoinsulinaemia and impaired glucose tolerance observed in the offspring of obese dams at 6 months of age.

The greatest reduction in insulin signalling protein levels was observed in IRS-1 which is well established as lying at the hub of insulin signalling [32]. Adipose tissue IRS-1 expression is reduced in humans and animals with type-2 diabetes [33] and in obese subjects [34]. Reduced IRS-1 expression in human and animal adipose tissue impairs downstream insulin signalling through the PI3K and AKT pathways, resulting in reduced insulin-stimulated glucose uptake [35]. Acute inhibition of IRS-1 in rats also leads to insulin resistance [36]. It is therefore likely that the reduction in IRS-1 in the offspring of obese dams observed here contributes to their development of insulin resistance.

There has been much attention in the programming field on the role of epigenetic mechanisms mediating the relationship between a suboptimal early environment and long-term health (reviewed in Barnes and Ozanne [37] and Galjaard [38]). These have primarily focused on programmed changes in DNA methylation and histone modifications in regulating transcription. However there are other non-genetic/epigenetic regulatory mechanisms that are operating in programming which are less well studied. For example, there are increasing instances in which programmed changes in protein expression are not accompanied by changes in transcript expression [22,39]. Hence, our observation of reduced adipose tissue IRS-1 protein levels without a parallel reduction in *IRS1* transcription suggests that the mechanism underlying its altered expression operates at the post-transcriptional level. Possible mechanisms include alterations in IRS-1 protein synthesis or degradation. Previous studies have shown that the protein levels of IRS-1 may be regulated by degradation through Ser307 phosphorylation [40]. In contrast, regulation of IRS-1 synthesis can occur through miRNA-mediated reductions in translation [41,42] and our findings support a role for such a mechanism through binding of *miR-126*.

MicroRNAs are short non-coding RNA molecules which function by binding specific sequences within the 3' untranslated region of target mRNAs and repressing mRNA translation [43]. Numerous miRNAs have been associated with obesity, insulin resistance and type-2 diabetes [44,45]. However to date, only a very limited number of studies have identified miRNAs as being involved in developmental programming. In the offspring of high fat diet fed dams, hepatic miRNAs whose predicted targets were proteins important for epigenetic regulation such as methylCpG binding domain proteins are differentially expressed [46]. More recently, we demonstrated that *miR-483* expression was increased in both rodents and humans in response to a suboptimal early nutrition and intrauterine growth restriction leading to a reduction in adipocyte expandability [39].

In the current study, we describe a novel observation that maternal diet-induced obesity leads to an increased expression of *miR-126* in adipose tissue. While *miR-126* has been shown previously to directly regulate IRS-1 translation in hepatocytes [42] and in HEK293 and MCF7 breast cancer cells [41], we have shown for the first time that IRS-1 is a potential target of *miR-126* in adipose tissue. A programmed increase in miR-126 in response to maternal diet-induced obesity therefore provides one mechanism by which IRS-1 may be silenced at the translational level. Although some studies show *miR-126* to be co-regulated with its host gene *Egfl7*, *miR-126* expression did not parallel that of *Egfl7* in adipose tissue in the current study. This could mean that they are independently regulated by maternal diet-induced obesity, either through the use of different promoters or through differences in stability. Crucially, we showed that the programmed loss of IRS-1 protein and increase in *miR-126* in adipose tissue resulting from maternal diet-induced obesity is maintained in primary adipocyte precursors isolated from epididymal fat from 8-week-old offspring, which were then expanded in culture and then differentiated into adipocytes. To confirm that the loss of IRS-1 was not driven by increased degradation, we applied a translational block in primary preadipocytes by treating with cycloheximide and showed that rate of IRS-1 decay was not affected by maternal diet-induced obesity. This suggests that the loss of IRS-1 is not driven by proteosomal degradation, and that it results from decreased protein synthesis, consistent with the observed increase in miR-126.

The maintenance of the programmed phenotype (i.e. decreased IRS-1 and increased *miR-126* in isolated preadipocytes differentiated *in vitro*) strongly suggests that the mechanism is cell autonomous and is retained following multiple rounds of cell division.

In conclusion, we have demonstrated that maternal diet-induced obesity leads to offspring insulin resistance prior to development of obesity. This is associated with reductions in key adipose tissue insulin signalling proteins including IRS-1 that is programmed at the translational level. These findings provide a mechanism by which maternal diet-induced obesity leads to increased risk of type-2 diabetes in the offspring. The adipose tissue insulin signalling proteins influenced by maternal obesity are very similar to those differentially expressed in low birth weight humans and animal models of foetal under-nutrition. This suggests that in response to a wide range of suboptimal early environmental conditions, common pathways are initiated which ultimately lead to increased risk of type-2 diabetes.

## Funding

This research was supported by the MRC Metabolic Diseases Unit (D.S. Fernandez-Twinn and S.E. Ozanne), the European Union's Seventh Framework Programme (FP7/2007-2013) project EarlyNutrition under Grant agreement no. 289346 (S.E. Ozanne), a scholarship from the Commonwealth Trust (A. Piekarz), the British Heart Foundation (M.S. Martin-Gronert and S.E. Ozanne), the Wellcome Trust (M.Z. Alfaradhi), the MRC Toxicology Unit (D. Ferland-McCollough) and a MRC Senior Fellowship (M. Bushell). D. E. Duque-Guimaraes is sponsored by a scholarship from the Brazilian Government - CNPq.

## Conflict of interest

None declared.

## Figures and Tables

**Table 1:** Maternal body composition at the end of lactation.

	**Control**	**Obese**
Lean mass (g)	21.7±0.7	20.2±0.9
% Lean mass	75.7±2.0	56.3±4.0⁎⁎⁎
Fat mass (g)	4.8±0.3	14.1±2.7⁎⁎⁎
% Fat mass	18.0±0.72	39.8±5.3⁎⁎⁎

⁎⁎⁎ *p*<0.001.

**Table 2:** Maternal serum metabolites at the end of lactation.

**Fed serum**	**Control**	**Obese**
Glucose (mmol/l)	9.4±0.5	11.77±1.04⁎
Triglycerides (mmol/l)	0.8±0.1	0.8±0.1
Free fatty acids (μmol/l)	410.8±45.6	796.4±130.0⁎
Cholesterol (mmol/l)	2.1±0.2	3.8±0.2⁎⁎⁎
LDL (mmol/l)	0.9±0.1	1.1±0.2
HDL (mmol/l)	1.0±0.1	1.7±0.1⁎⁎⁎

⁎ *p*<0.005.

⁎⁎⁎ *p*<0.001.
