# Supplementary material for: Downregulation of IRS-1 in adipose tissue of offspring of obese mice is programmed cell-autonomously through post-transcriptional mechanisms
Source: Mol Metab. 2014 Jan 20;3(3):325–33. doi: 10.1016/j.molmet.2014.01.007 (PMC3986586; doi:10.1016/j.molmet.2014.01.007)
Supplement: Supplementary file 1 — Supplementary material [file mmc1.docx]

**Supplementary Table 1**: Forward and reverse primer sequences

| Primer | Forward 5’-3’ | Reverse 5’-3’ |
| --- | --- | --- |
| IRβ | cagccggatgggccaatggga | ctcgtccggcacgtacacagaa |
| IRS1 | tcctatcccgaagagggtct | tgggcatatagccatcatca |
| p110β | gcgcggggcagttcatcttctaa | gaggcatgatagggcggaagca |
| p85α | gccaaggaaactgtcgcacaca | ggggcagtgctggtggatccat |
| AKT1 | atgaacgacgtagccattgtg | ttgtagccaataaaggtgccat |
| AKT2 | ccacgacccaacacctttgt | gatagcccgcatccactcttc |
| HPRT | gctcgagatgtcatgaaggagat | aaagaacttatagccccccttga |
| Beta-Actin | ttcaacaccccagccatgta | tgtggtacgaccagaggcatac |
| Cyclophilin | agggtggtgactttacacgc | gatgccaggacctgtatgct |
| PABP1 | atgttgggtgaacggctgtttcct | gagcggagagactctggagactcg |
