# Supplementary material for: Downregulation of IRS-1 in adipose tissue of offspring of obese mice is programmed cell-autonomously through post-transcriptional mechanisms
Source: Mol Metab. 2014 Jan 20;3(3):325–33. doi: 10.1016/j.molmet.2014.01.007 (PMC3986586; doi:10.1016/j.molmet.2014.01.007)
Supplement: Supplementary file 2 — Supplementary material [file mmc2.docx]

**Supplementary Table 2**: Body composition of 8-week-old male offspring

|  | **Control** | **Mat-Ob** |
| --- | --- | --- |
| Body weight (g) | 24.0 ± 0.4 | 23.5 ± 0.7 |
| Lean mass (g) | 18.5 ± 0.4 | 17.4 ±0.4 |
| Fat mass (g) | 3.7 ± 0.1 | 3.8 ± 0.2 |
| % Lean mass | 83.3 ± 0.6 | 82.2 ± 1.0 |
| % Fat mass | 16.8 ± 0.5 | 17.7 ± 1.0 |
| % Bone density | 0.04 ± 0.0007 | 0.04 ±0.0008 |
